# Lights triggered differential accumulation of antioxidant and antidiabetic secondary metabolites in callus culture of *Eclipta alba* L.

**DOI:** 10.1371/journal.pone.0233963

**Published:** 2020-06-12

**Authors:** Razia Khurshid, Muhammad Asad Ullah, Duangjai Tungmunnithum, Samantha Drouet, Muzamil Shah, Afifa Zaeem, Safia Hameed, Christophe Hano, Bilal Haider Abbasi

**Affiliations:** 1 Department of Biotechnology, Quaid-i-Azam University, Islamabad, Pakistan; 2 Laboratoire de Biologie des Ligneux et des Grandes Cultures (LBLGC), INRA USC1328, Université d’Orléans, Orléans, France; 3 Department of Pharmaceutical Botany, Faculty of Pharmacy, Mahidol University, Rajathevi, Bangkok, Thailand; 4 COSM’ACTIFS, Bioactifs et Cosmétiques, CNRS GDR3711, Orléans, France; National University of Kaohsiung, TAIWAN

## Abstract

*Eclipta alba* L., also known as false daisy, is well known and commercially attractive plant with excellent hepatotoxic and antidiabetic activities. Light is considered a key modulator in plant morphogenesis and survival by regulating important physiological cascades. Current study was carried out to investigate growth and developmental aspects of *E*. *alba* under differential effect of multispectral lights. *In vitro* derived callus culture of *E*. *alba* was exposed to multispectral monochromatic lights under controlled aseptic conditions. Maximum dry weight was recorded in culture grown under red light (11.2 g/L) whereas negative effect was observed under exposure of yellow light on callus growth (4.87 g/L). Furthermore, red light significantly enhanced phenolics and flavonoids content (TPC: 57.8 mg/g, TFC: 11.1 mg/g) in callus cultures compared to rest of lights. HPLC analysis further confirmed highest accumulation of four major compounds i.e. coumarin (1.26 mg/g), eclalbatin (5.00 mg/g), wedelolactone (32.54 mg/g) and demethylwedelolactone (23.67 mg/g) and two minor compounds (*β*-amyrin: 0.38 mg/g, luteolin: 0.39 mg/g) in red light treated culture whereas stigmasterol was found optimum (0.22 mg/g) under blue light. *In vitro* based biological activities including antioxidant, antidiabetic and lipase inhibitory assays showed optimum values in cultures exposed to red light, suggesting crucial role of these phytochemicals in the enhancement of the therapeutic potential of *E*. *alba*. These results clearly revealed that the use of multispectral lights in *in vitro* cultures could be an effective strategy for enhanced production of phytochemicals.

## Introduction

Plants have been the source of food, feed and medicine for centuries in almost all cultures around the world. Medicinal properties of herbs have been exploited exponentially to treat plethora of diseases in past centuries [1]. Modern era of pharmaceutical industry emphasizes on isolation of active individual compounds, playing key role in treating specific disease [2]. One of such medicinally important plants is *Eclipta alba* L., commonly known as false daisy. *E*. *alba* is herbaceous, annual plant which belongs to family *Asteraceae* [3]. It is located in world’s tropical and subtropical areas, fostering over vast regions of land [4]. Almost all parts of *E*. *alba* have been identified with medicinal properties, especially roots were employed as disinfectant for wounds, as laxative and ulcer treatment in livestock. Extract of *E*. *alba* have been used as tonic for blood vessel clearance in spleen and liver whereas antihepatotoxic activity have previously been reported in various studies to treat liver diseases [5–7]. Shoot extract of *E*. *alba* was found effective as bronchodilator, anti-inflammatory and antimicrobial agent [8]. *E*. *alba* possess wide range of industrially important phytochemicals that are responsible for its antianaphylactic, antidiabetic and antioxidant properties [9]. Some of these compounds have been utilized commercially in drug named Abana [10]. Among various secondary metabolites, coumestan-type compounds are present as majority constituents in *E*. *alba* phytochemical profile. Two of coumestan-type compounds i.e. wedelolactone and demethylwedelolactone have been isolated and identified in *E*. *alba*. These compounds are known to have significant antihepatotoxic activity, in curing hepatitis and liver cirrhosis [11]. Another novel triterpene saponin called eclalbatin have been isolated from *E*. *alba* along with α-amyrin, β-amyrin, ursolic acid and oleanolic acid [12].

Production of these compounds is usually enhanced under the influence of external stress or environmental fluctuations to cope with drastic effects of stress. Application of stress in *in vitro* conditions can be beneficial for scaling up the yield of these phytochemicals. One of the most important and efficient strategies is “Elicitation”, in which metabolic pathways are triggered by incorporating agents (elicitors) for optimum production of secondary metabolites [13, 14]. Light is an effective abiotic elicitor that influences plant photosynthesis process, development and morphogenesis [15, 16]. Light play vital role in regulating primary as well as secondary metabolism to help achieve optimum growth [17–19]. Multiple studies reported the direct stimulation of secondary metabolites production in the presence of multispectral monochromatic lights [17, 20–22]. However, the synergistic/antagonistic role of light is greatly influenced by light intensity, quality, exposure time, plant species and culture type [23].

Current study was designed to evaluate differential accumulation of phytochemicals in *E*. *alba* callus culture in response to multispectral lights. Phytochemical quantification was carried out by high performance liquid chromatography and different antioxidant, antidiabetic and lipase inhibition activities were performed by employing *in vitro* based assays.

## Material and methods

### *In vitro* seed germination and callus induction

*E*. *alba* seeds were obtained from PCCL (Plant Cell Culture Laboratory), Quaid-i-Azam University Islamabad, Pakistan and surface sterilized by protocol proposed of Abbasi et al. [24] with slight adjustments. Seeds were dipped for 1 min into 0.1% mercuric chloride solution, followed by 70% ethanol treatment for 2 min. Autoclaved dH_2_O was used to thoroughly wash seeds for 3 times prior to inoculation on Murashige and Skoog (MS0) media. MS [25] media additionally fortified with solidifying agent (0.8% agar) and carbon source (3% sucrose) was used to inoculate seeds and medium pH was set around 5.6–5.7. Prior to seed inoculation, media was autoclaved at 121°C temperature for 20 min. Constant temperature (25 ± 2°C) and light (8 dark/16 light) conditions were maintained in growth room for proper germination of seeds. Stem explant was taken from 28 days old plantlets and placed in hormonally optimized MS media, supplemented with 3.0 mg/L BAP (6-benzylaminopurine) and 1.0 mg/L NAA (α-naphthalene acetic acid) as previously studied by Khurshid et al. [26]. Explant inoculated culture flasks were placed under aseptic conditions in growth room (Photoperiod: 8 dark/16 light and Temperature: 25 ± 2°C) for 4 weeks. To achieve proper biomass, callus was sub-cultured after every 28 days on respective hormonal media.

### Callus exposure to differential lights

Total of seven light treatments were employed to study biosynthesis of secondary metabolites in *E*. *alba* callus culture. These lights were of varying wavelengths i.e.

- Yellow LEDs (570 nm, 24-hrs)
- Green LEDs (510 nm, 24-hrs)
- White Light (400–700 nm, 24-hrs)
- Blue LEDs (460 nm, 24-hrs)
- Red LEDs (660 nm, 24-hrs)
- Cool-White florescent tubes (400–700 nm, 16/8h light/dark, Photoperiod, as control)
- Dark (24-hrs)

Callus (1g), obtained from subsequent sub-cultures, was inoculated on hormonally optimized media (40 ml) and placed under each light for 28 day with light intensity of 40–50 μmol m^−2^s^−1^. Light intensity was measured and optimized by Advanced Lux meter (SU10, Jeio-tech). Growth parameters and phytochemical analysis were carried out after 4 weeks of light exposure to callus.

### Growth parameters and extraction of callus samples

After 28 days of light exposure, calli were removed from media onto Whatman filter paper for separation of extra water contents. Fresh weight of calli were measured and further placed in incubator at 45°C for 24 hours to measure dry weight. To carryout phytochemical and antioxidant analysis, dried calli were subjected to extraction protocol proposed by Zahir et al. [27]. Callus powder (0.1g) was homogenized with methanol (500 μl) by 30 min of sonication and 15 min of vortexing. Prior to centrifugation (10min, 15000rpm), extraction procedure was repeated twice. After centrifugation, pallet was discarded, and supernatant was stored at 4 °C.

### Phytochemical analysis

#### Phenolic and flavonoid contents

Total phenolic contents (TPC) were evaluated by employing Folin-Ciocalteu (FC) reagent according to Singleton and Rossi [28]. FC reagent (90 μl) was mixed with methanol mediated callus extract (20 μl) for each treatment along with Na_2_CO_3_ solution (90 μl) in 96 welled micro-plate. Absorbance reading was measured using micro-plate reader (Thermo Scientific Multiskan GO), after 5 min incubation, at 630 nm wavelength. Gallic acid was used as standard and phenolic contents were expressed as GAE/g in equivalent of gallic acid. Phenolic production was calculated by following formula,

TotalPhenolicProduction(TPP:mg/L)=TPC(mg/g)×dryweight(g/L) (1)

For total flavonoid contents (TFC) evaluation, Aluminum chloride colorimetric method was employed according to Ahmed at al. [29]. Aluminum chloride (10 μl) was mixed in methanol derived callus extract (20 μl) and 10 μl potassium acetate in 96 welled micro-plate, followed by 160 μl of dH_2_O. Absorbance reading was measured using micro-plate reader (Thermo Scientific Multiskan GO), at 415 nm wavelength after 30 min incubation. Quercetin was used as standard and flavonoid contents were expressed as QE/g in equivalent of quercetin. Flavonoid production was calculated by following formula,

TotalFlavonoidProduction(TFP:mg/L)=TFC(mg/g)×dryweight(g/L) (2)

#### HPLC quantification of metabolites

Quantification of the major *E*. *alba* callus phytochemicals was performed according to Khurshid et al. [26]. Briefly, RP-HPLC was used for separation and quantification, equipped with chromatographic Varian liquid system, autosampler Varian Prostar 410, Metachem Degasit, Photodiode Array Detector Varian Prostar 335, Varian Prostar 230 pump. Galaxie software 1.9.3.2 version was used to control the system. At 35 °C separation temperature, Purospher (Merck) RP-18 column was used to perform separation. Mobile phase consisted of two solvents i.e. solvent A (acetic acid (0.2%) in water) and solvent B (acetic acid (0.2%) in methanol). At 0.8 ml/min flow rate during non-linear gradient run, mobile phase composition varied from 0 to 40 min of A–B: 90:10 to 30:70 (v/v), from 41 to 50 min of A–B: 30:70 to 0:100 (v/v), and A–B: 0:100 (v/v) from 51 to 60 min. Compounds of *E*. *alba* were detected at 204 nm. Standards (β-amyrin, stigmasterol, coumarin and wedelolactone) were purchased from Sigma-Aldrich. Quantification of eclalbatin and desmethylwedelolacetone were done using the calibration curves of β-amyrin and wedelolactone respectively and were expressed as β-amyrin and wedelolactone equivalent concentration respectively.

### Antioxidant activity of callus extracts

#### DPPH scavenging activity

2,2-diphenyl-1-picrylhydrazyl reagent (DPPH) was employed to determine antioxidant activity according to Abbasi et al. [24]. Briefly, 180 μl DPPH solution was thoroughly mixed in methanol derived calli extract (20 μl) for each treatment in 96 welled micro-plate and kept in dark for one hour. Absorbance reading was measured after incubation period at 517 nm wavelength. Free radical scavenging activity was calculated using following formula

DPPH%=100×(1−AE/AD)

Here AD: absorbance value of DPPH solution in the absence of extract whereas AE expresses in the presence of calli extract.

#### Ferric reducing antioxidant power (FRAP) assay

Benzie and Strain [30] protocol was employed to study FRAP activity of light treated calli extracts. FRAP solution is generally composed of three major components i.e. FeCl_3_.6H_2_O (20 mM), acetate buffer (300 mM, pH 3.6) and TPTZ (10 mM) in ratio of 1:10:1 (v/v/v). Briefly, 190 μl FRAP solution was thoroughly mixed with 10 μl calli extracts and incubated for 15 min at room temperature in 96 welled micro-plate, followed by absorbance measurement at 630 nm. The absorbance was expressed as Trolox C equivalent antioxidant capacity (TEAC).

#### Antioxidant ABTS assay

Antioxidant potential of calli extracts was evaluated according to Tagliazucchi et al. [31] method using ABTS solution (2,2-azinobis (3-ethylbenzthiazoline-6-sulphonic acid)). Briefly, potassium persulphate (2.45 mM) was mixed in equal proportion with 7 mM ABTS salt to prepare ABTS solution. The prepared solution was kept in dark for 16 hours. Before mixing calli extract, OD of solution adjusted to 0.7 at 734 nm. Reaction mixture containing extract and ABTS solution was kept in dark at room temperature for 15 min. Absorbance was taken at 734 nm and values were expressed Trolox C equivalent antioxidant capacity (TEAC).

### Antidiabetic activity of callus extracts

#### α-glucosidase inhibition

Rat intestinal acetone powder, purchase from Sigma Aldrich, was used to obtain partially purified α-glucosidase. Enzymatic immobilization was done according to Hano et al. [32] on CNBr-activated sepharose 4B. Hano et al. [32] proposed chromogenic method was employed to evaluate enzymatic activity using end capped 0.45 μm polyethylene filter column. Briefly, intestinal fluid (1mL) was used to perform assay containing 4-nitrophenyl-α-D-glucopyranoside (5mM, 4NPG; Sigma). After half hour incubation at room temperature, solution was column filtered to stop reaction and sodium carbonate (1M) solution was added in equal volume. Increase in absorbance value compared to blank at 405 nm was used to determine enzymatic activity. The % enzyme inhibition for each sample was calculated by subtraction of absorbance values in the presence and absence of calli extracts.

#### α-amylase inhibition

The α-amylase from porcine pancreas was purchased from Sigma. Hano et al. [32] described chromogenic method was employed to evaluate soluble α-amylase activity. In brief, phosphate buffer (0.1 M, pH6.8) was used to prepare enzyme at 1u/mL concentration and thoroughly mixed in 5mM of 4-nitrophenyl-α-D-maltopentaoside (s4NPM; Sigma). After half hour incubation at room temperature, solution was column filtered to stop reaction and sodium carbonate (1M) solution was added in equal volume. Increase in absorbance value compared to blank at 405 nm was used to determine enzymatic activity. The % enzyme inhibition for each sample was calculated by subtraction of absorbance values in the presence and absence of calli extracts.

#### Anti-AGEs formation activity

Kaewseejan and Siriamornpun [33] method was used to determine inhibitory potential of AGEs (advanced glycation end products) formation. Extract preparation was carried out at a concentration of 50μg/ml in DMSO mixed with 0.5 M glucose and 20 mg/ml BSA solution, both were prepared in phosphate buffer and 1 ml of 0.1 M phosphate buffer containing 0.02% (w/v) sodium azide at pH 7.4. The level of AGEs formation was determined after 5 days incubation period at room temperature in the dark using fluorescent spectrometer (Bio rad Versa Fluor) with an excitation wavelength set at 330 nm and emission wavelength set at 410 nm. The inhibitory capacity of AGEs formation was expressed as % inhibition relative to control (equal volume of DMSO).

### Lipase activity

Lipase inhibitory potential of calli extracts was evaluated according to Chen et al. [34]. Briefly, centrifuge tube (1.5 ml) was used to mix 100μl pancreatic lipase solution (0.5 mg/ml) with 20μl extract, followed by addition of 900μl Tris–HCl buffer (pH 7.5). A pNPB solution (100ul, 10mmol/l) was thoroughly mixed and oscillated after incubated of reaction mixture at room temperature for 15 min and transferred to micro-plate (96 well). Change in absorbance value (change rate K) was determined using micro-plate reader over 15 min at 400nm wavelength. The inhibition rate was calculated as follows:

Inhibitionrate(%)=(Knormalvalue−Kexperimentalvalue)/Knormalvalue100%.

### Statistical analysis

Whole experiment was repeated twice and in triplicates. Data on standard error and mean values was calculated using Microsoft excel program and graphs were generated with Origin software (8.5). Data was expressed in terms of mean ± SE. Tukey’s multiple comparisons test was employed for calculating significant differences. One-way analysis of variance (ANOVA) with significant difference *p* < 0.05 was used to compare the means of different treatments. Pearson’s correlation coefficients were obtained using XL-STAT 2020 (Addinsoft, Paris, France).

## Results and discussion

### Differential lights influence on biomass accumulation

**Fig 1:**
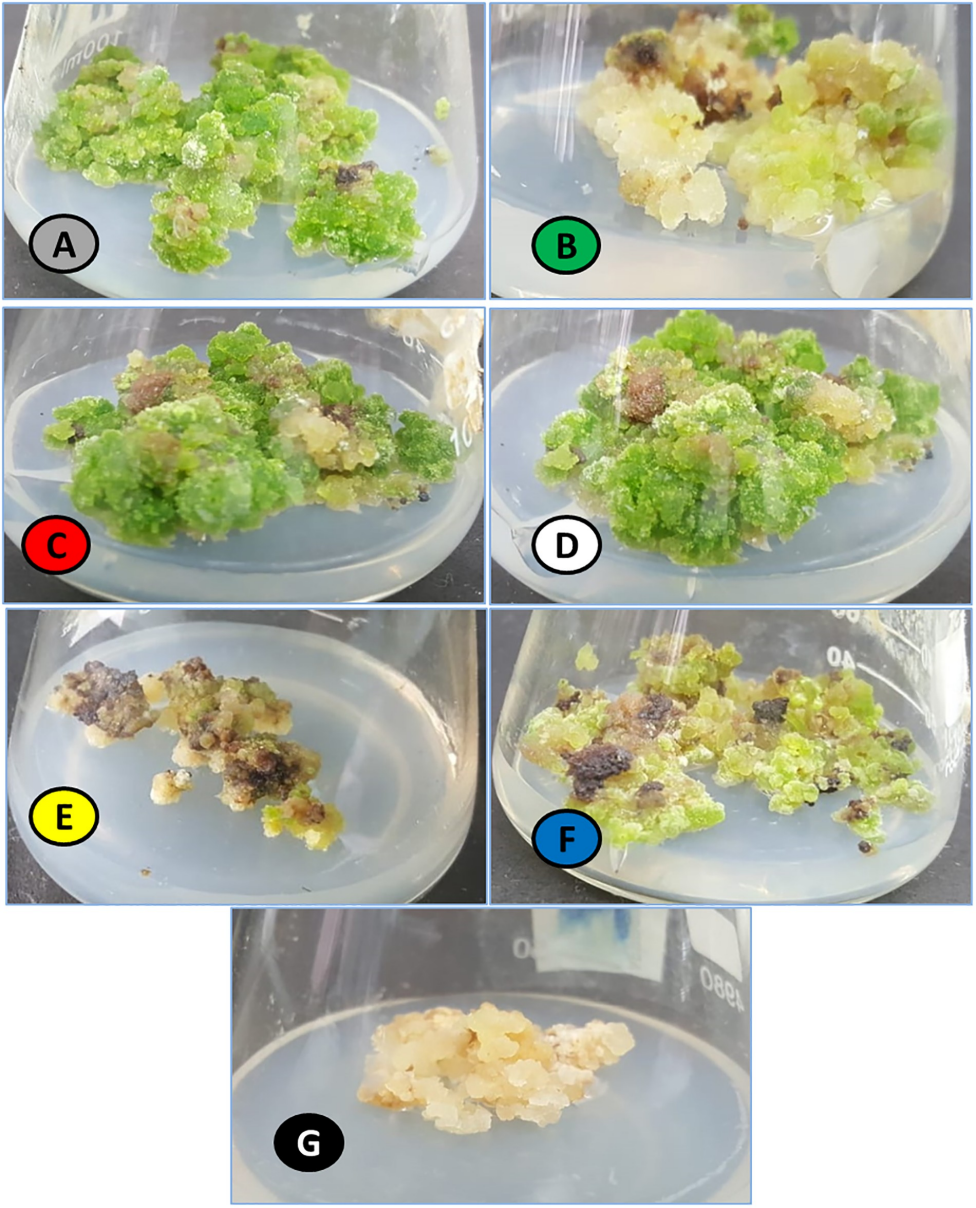
Morphological variation in callus culture placed under different monochromatic lights. (A = . Control; B = Green; C = Red; D = White; E = Yellow; F = Blue; G = Dark).

**Fig 2:**
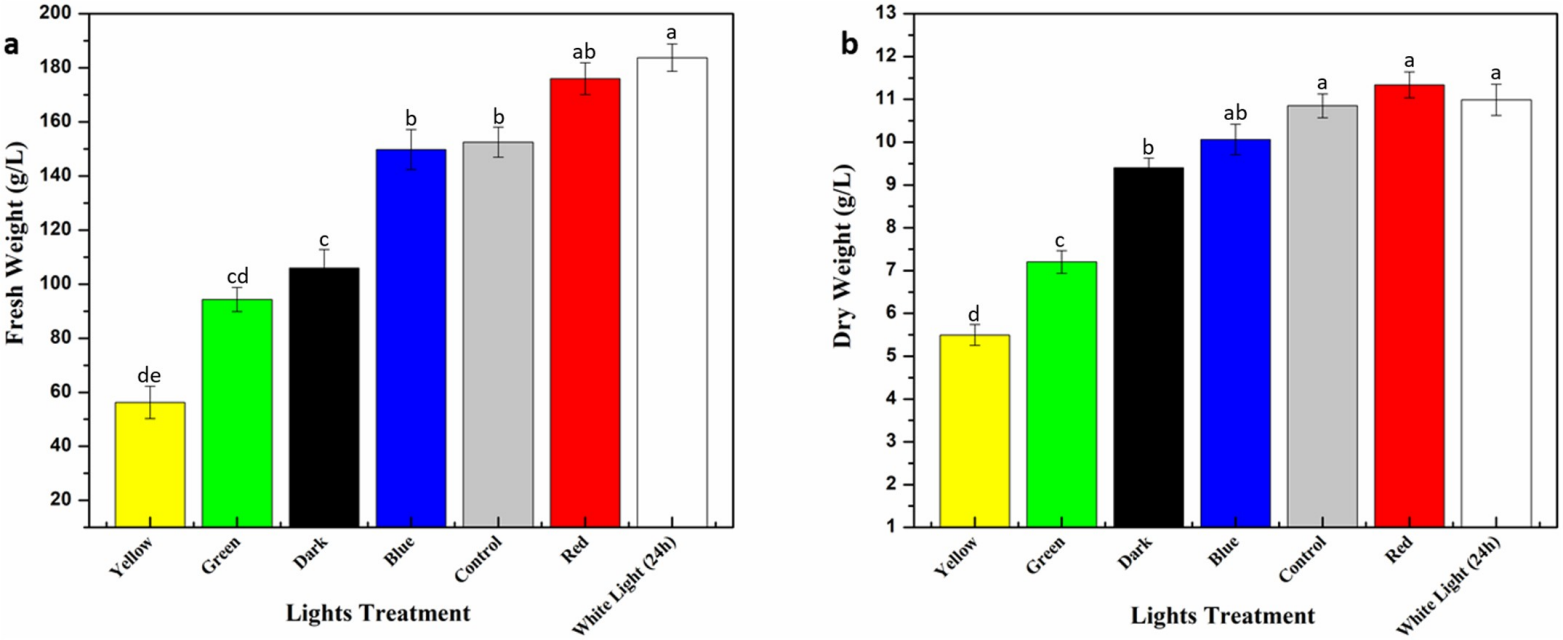
**a)** Fresh biomass accumulation of callus culture **b)** Dry weight accumulation of callus culture in response to monochromatic lights.

In current study, monochromatic light’s effect was investigated for enhanced biomass accumulation in callus culture of *Eclipta alba*. Callus culture maintained at 3.0 mg/L BAP and 1.0 mg/L NAA hormonal treatment was placed under different lights whereas callus under photoperiod conditions (16h light/8h dark) was considered as control (Fig 1). Highest fresh weight (FW: 183.7 g/L) was observed in response to white light (24-hrs), followed by red (FW: 176.1 g/L) and control (FW: 152.5 g/L) (Fig 2a) whereas optimum dry weight (DW: 11.2 g/L) was accumulated under red light (Fig 2b). Maximum fresh weight under white light could possibly be due to higher accumulation of water contents in cells. These results basically depict stimulatory effect of red light on enhanced biomass accumulation in callus culture of *Eclipta alba*. The possible reason behind this increase in biomass accumulation might be conversion of phytochrome into its active form Pfr when illuminated with red light, which may have increased synthesis and activities of growth-related enzymes [35]. Our results are in agreement with Yu et al. [36], where maximum biomass accumulation under red light in hairy root culture of *Panax ginseng* was recorded. Likewise, Kapoor et al. [37] also suggested that cultures irradiated with red light resulted in highest accumulation of biomass in *in-vitro* cultures of *Rhodiola imbricate*, which are in harmony with our findings. Yellow light significantly reduced biomass accumulation (FW: 56.2 g/L, DW: 4.87 g/L) compared to rest of lights. Effect of light quality varies according to plant species, cell type and intensity of light [38]. On contrary, blue and white light has previously been reported to have significant impact on biomass production in different plant species [15, 23, 39, 40].

### Phenolic and flavonoid accumulation in response to light exposure

**Fig 3:**
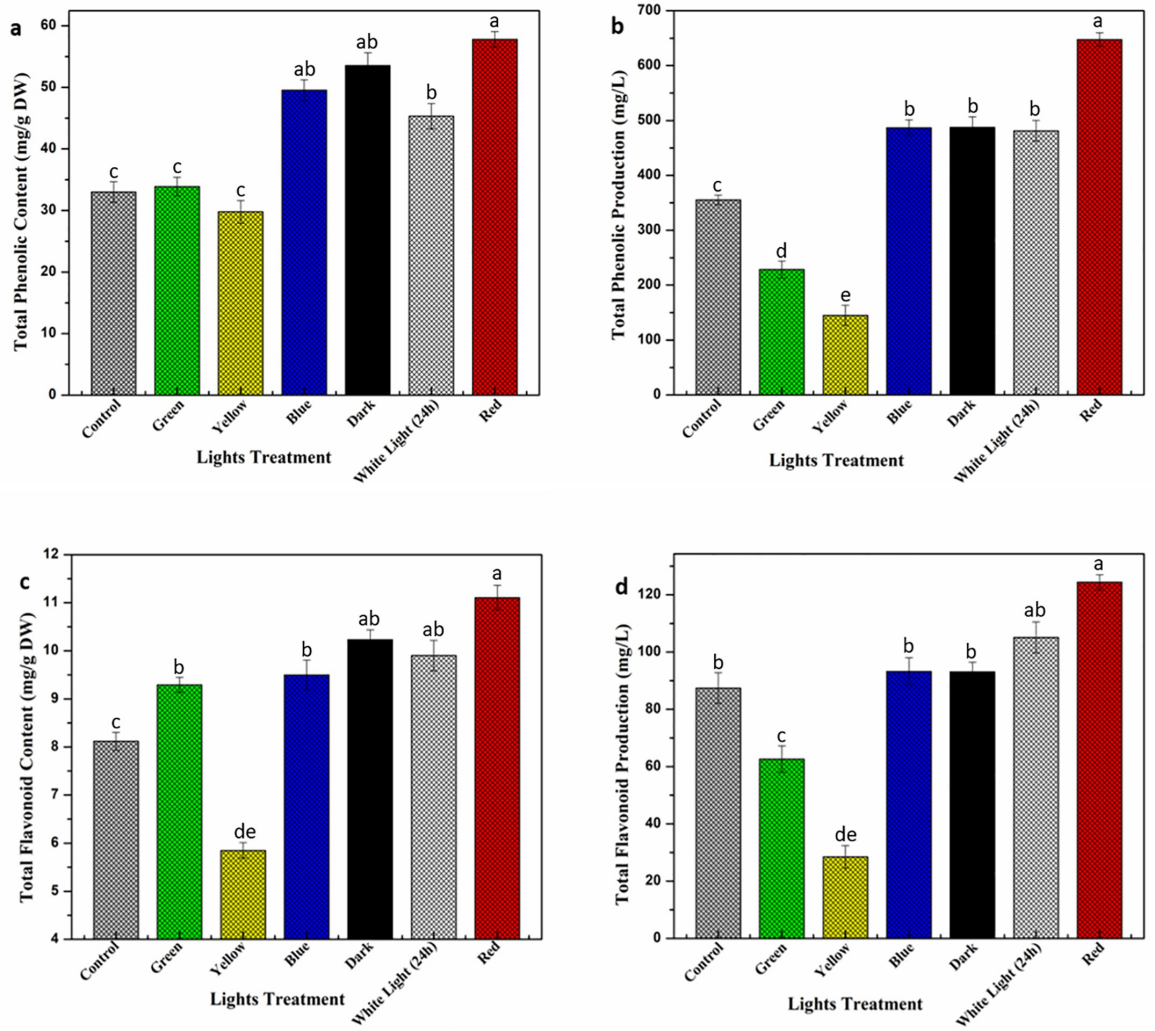
Differential effect of lights on a) TPC (mg/g DW): b) TPP (mg/L): c) TFC (mg/g DW): d) TFP (mg/L) accumulation in callus cultures. Error bar represents means deviation of triplicates.

Plants tend to produce a variety of phytochemical in response to environmental stress. These phytochemical contents play vital role to ensure proper growth and development under harsh conditions [41, 42]. Light stress has extensively been used in the past for enhanced production of plant secondary compounds [18, 43]. Present study investigates the accumulation of pharmaceutically important phytochemicals (phenolic and flavonoids) in callus culture of *E*. *alba*, exposed to multispectral lights. Optimum phenolic contents (TPC: 57.81 mg/g) and phenolic production (TPP: 647.4 mg/L) values were recorded in response to red light, followed by dark (TPC: 53.55 mg/L, TPP: 487.2 mg/L) and blue (TPC: 45.54 mg/g, TPP: 486.3 mg/L). Almost 2-fold increase was observed in phenolics accumulation under red light compared to control (Fig 3a and 3b). Increase level of cytokinin under red light could possibly be the cause of enhanced phenolics accumulation but the exact phenomena is still not clearly understood [44]. Similar results were noted in flavonoids accumulation in response to red light (TFC: 11.1 mg/g, TFP: 124.3 mg/L) compared to control (TFC: 8.1 mg/g, TFP: 87.4 mg/L) suggesting a positive correlation in phenolics and flavonoids under multispectral lights (Fig 3c and 3d). Yellow and green light significantly reduced phytochemical contents in callus culture of *E*. *alba* (Fig 3). Previously, Younas et al. [45] reported enhanced silymarin accumulation under red light exposure whereas yellow light significantly reduced silymarin contents in callus culture of *Silybum marianum L*. Red light has previously been reported for enhanced phytochemical accumulation in *Myrtus communis* and *Rehmannia glutinosa in vitro* cultures [46, 47]. However, in contrast to our results, Gupta and Kharmakar [48] and Fazal et al. [43] reported enhanced production of phytochemicals under blue light in *S*. *chirata* shoot culture and *P*. *vulgaris* callus culture respectively, which suggest that light effect on plants varies depending upon plant species, culture type and light quality [49].

### Lights effect on *in vitro* antioxidant activities of *E*. *alba*

**Fig 4:**
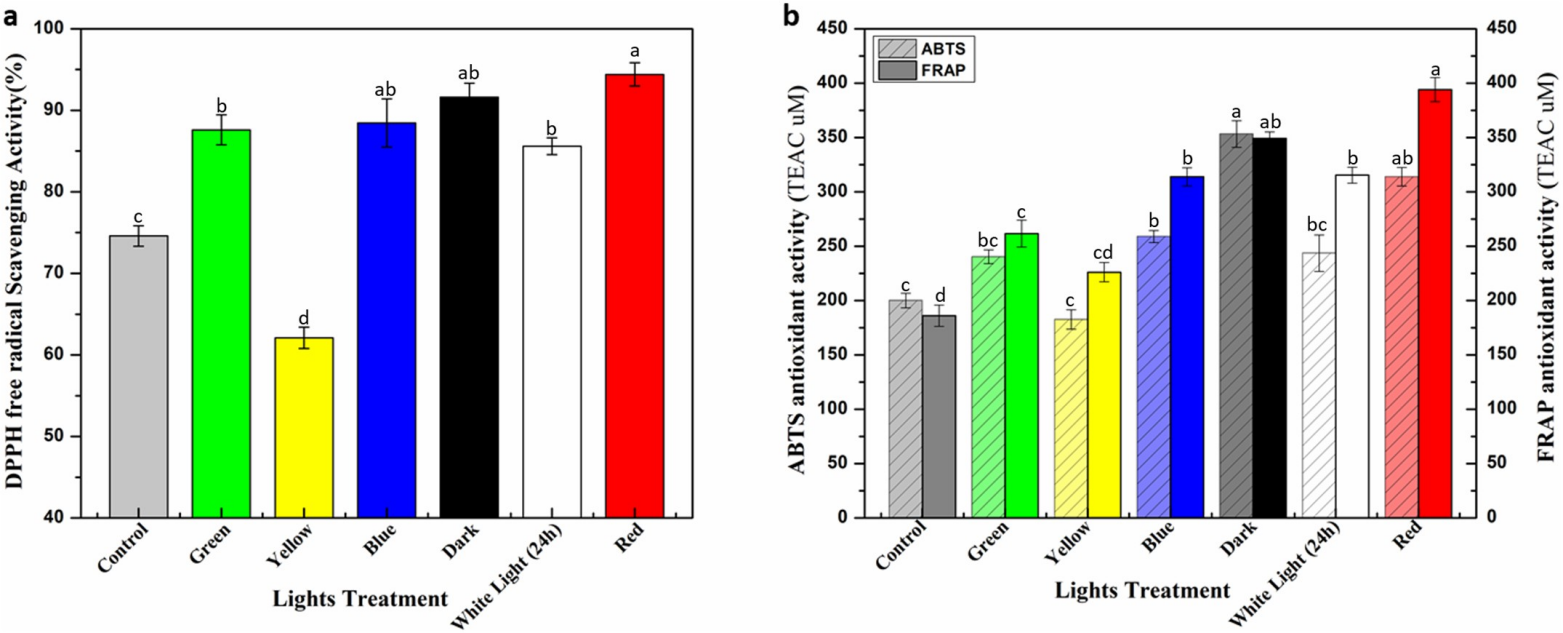
*In vitro* antioxidant activities of callus culture of *E*. *alba* a) DPPH (%), b) ABTS and FRAP assay (TEAC: Trolox C equivalent antioxidant activity, expressed in μM.

Environmental stress pushes plants to produce highly reactive oxygen free radicals during metabolic reactions which hinder growth, development and physiological processes [50–52]. Plants have protective mechanism in place to combat these reactive oxygen species (ROS) by synthesizing a wide range of secondary metabolites such as phenolic acids, flavonoids and terpenoids etc. Protective effect of these phytochemicals has been studied in the past for mitigation of oxidative damage of ROS [53–55]. Antioxidant activity in response to light stress was evaluated in current study by employing *in vitro* antioxidant assays, mainly DPPH (2,2-diphenyl-1-picrylhydrazyl), ABTS (2,2-azinobis (3-ethylbenzthiazoline-6-sulphonic acid)) and FRAP (Ferric Reducing Antioxidant Power) assay. Highest DPPH antioxidant activity (94.4%) was recorded in calli extracts placed under red light, followed by dark (91.6%) and blue light (88.4%). Yellow light negatively affected DPPH activity (62.1%) as compared to control (74.6%) (Fig 4a). Our findings are in agreement with previous study of Younas et al. [45] stating the dominating effects of red light on antioxidant activity. Relatively similar results were observed for ABTS and FRAP antioxidant assays in response to light stress on callus culture of *E*. *alba*. Red light treated cultures showed 2-fold increase in FRAP activity (394.04 μM) as compared to control (186.09 μM). However, maximum ABTS antioxidant activity was recorded in dark (353.2 μM), followed by red light (313.8 μM) (Fig 4b). Lowest ABTS activity (182.6 μM) was observed in yellow light treated callus cultures compared to control (200.07 μM). An obvious correlation in phytochemical accumulation and antioxidant potential simply suggest the regulatory role of these precious metabolites in shaping plant physiology and development [56, 57]. Enhanced phytochemical biosynthesis could possibly be the sole reason behind elevated antioxidant activities in callus culture of *E*. *alba* as reported in multiple studies [58–60].

### Lights influence on biological activities of *E*. *alba* callus cultures

**Fig 5:**
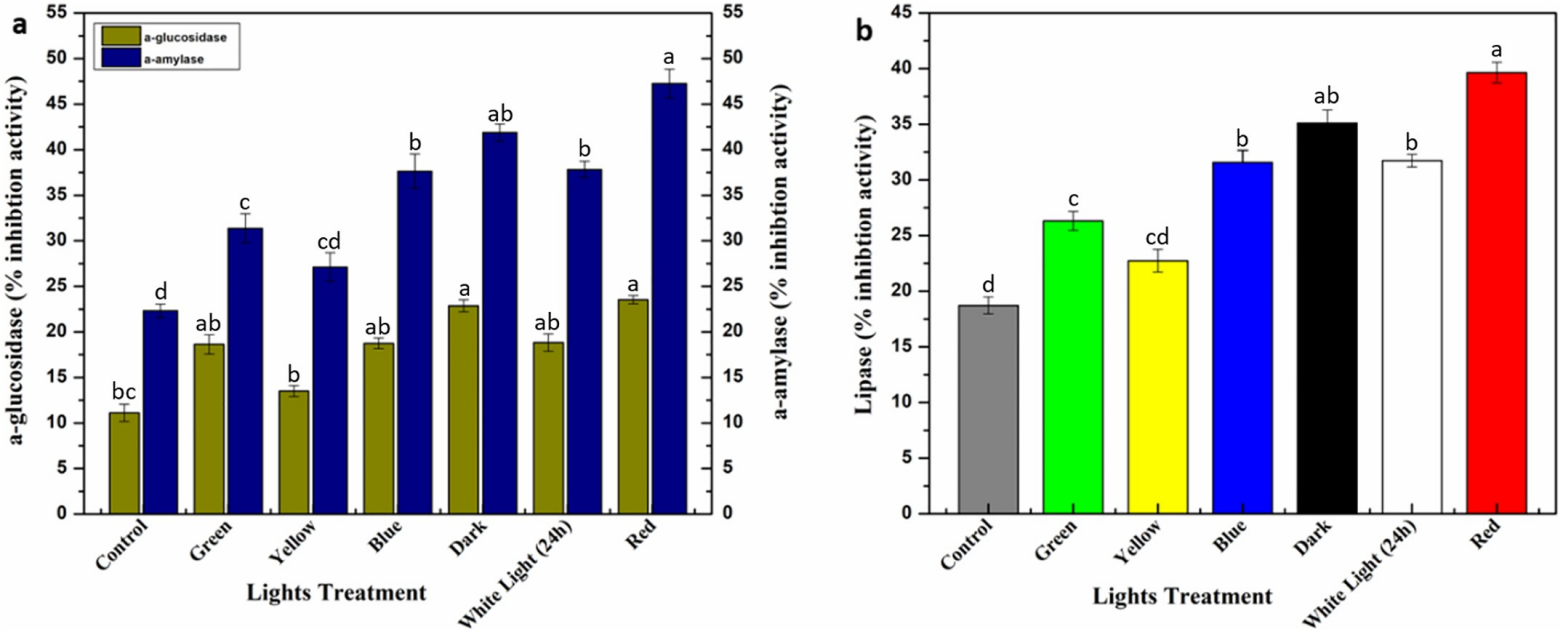
Differential effect of multispectral lights on a) Antidiabetic Activity (% inhibition) and b) Lipase Activity (% inhibition) in callus cultures of *E*. *alba*.

Diabetes and related complications around the world are major contributing reasons of morbidity and mortality [61, 62]. Plants possess huge potential as antidiabetic agents by preventing hyperglycemic conditions in diabetic patients. Antidiabetic potential of *E*. *alba* was evaluated in current study against multispectral lights using *in vitro* callus culture as model system. Two enzymes i.e. α-glucosidase and α-amylase play vital role in development of complications in diabetic patients (by degrading carbohydrate more than the patients can handle) and could be controlled by inhibiting their activity. Red light significantly enhanced α-glucosidase inhibition (23.54%) activity, followed by dark (22.86%) compared to control (11.12%). Similarly, a 2-fold increase in α-amylase inhibition activity (47.26%) by red light was observed, followed by dark (41.88%) as compared to control (22.32%) (Fig 5a). Overall, antidiabetic potential of *E*. *alba* callus extracts was mainly due to inhibition of α-amylase activity compared α-glucosidase as previously observed in *Linum usitatissimum* L. seed extract by Hano et al. [32]. Phytochemical production and enhanced antidiabetic activities suggested a positive correlation in response to red light in callus culture of *E*. *alba*. This is the first study determining lights application as an abiotic elicitor for antidiabetic activities of *E*. *alba*. During hyperglycemic conditions, diabetic patients develop several complications including formation of AGEs (Advance glycation end products) [63]. Therefore, inhibition potential of calli extracts against AGEs formation (pentosidine- and vesperlysine-like) was evaluated under monochromatic lights. Red light directly influenced the inhibitory potential of AGEs formation (pentosidine-like: 66.36%, vesperlysine-like: 42.66%), followed by dark, however, white and blue light showed similar results (Table 1). These results are directly in accordance with phytochemical accumulation, suggesting that phytochemicals can effectively be developed into therapeutic inhibitors to overcome diabetes related complications [64]. Obesity, like diabetes, has emerged as largest chronic disease according to WHO standards with adult death rate of 3.4 million/year [34, 65, 66]. Obesity can be controlled by inhibiting pancreatic lipase activity, an enzyme responsible for 50–70% fat decomposition [34]. Lipase inhibition potential of *E*. *alba* calli extracts was also evaluated in current study. Significant effect of lipase inhibitory activity was recorded under different monochromatic light. Calli placed under red light showed 2-fold increase in inhibition of lipase activity (39.6%) than control (18.72%) (Fig 5b). In general, monochromatic lights were proved to be an effective stimulus for enhanced biological activities of calli extracts by triggering phytochemical biosynthesis.

**Table 1:** % inhibition of advance glycation end products formation (AGEs) in callus culture of *E*. *alba*. Values represent means ± standard errors from triplicates.

Lights Treatment	Inhibition of Advanced Glycation End Products Formation (AGEs)	
	Vesperlysine-like AGEs (% Inhibition)	Pentosidine-like AGEs (% Inhibition)
**Control**	26.77 ± 1.942	36.89 ± 2.339
**Green**	32.54 ± 0.943	47.54 ± 1.994
**Yellow**	29.83 ± 2.266	42.53 ± 1.338
**Blue**	36.53 ± 1.053	54.94 ± 0.951
**Dark**	39.23 ± 0.683	59.94 ± 1.031
**White Light (24h)**	36.65 ± 1.395	55.16 ± 0.499
**Red**	42.66 ± 2.039	66.36 ± 2.045

### Quantification of phytochemicals *via* HPLC

**Fig 6:**
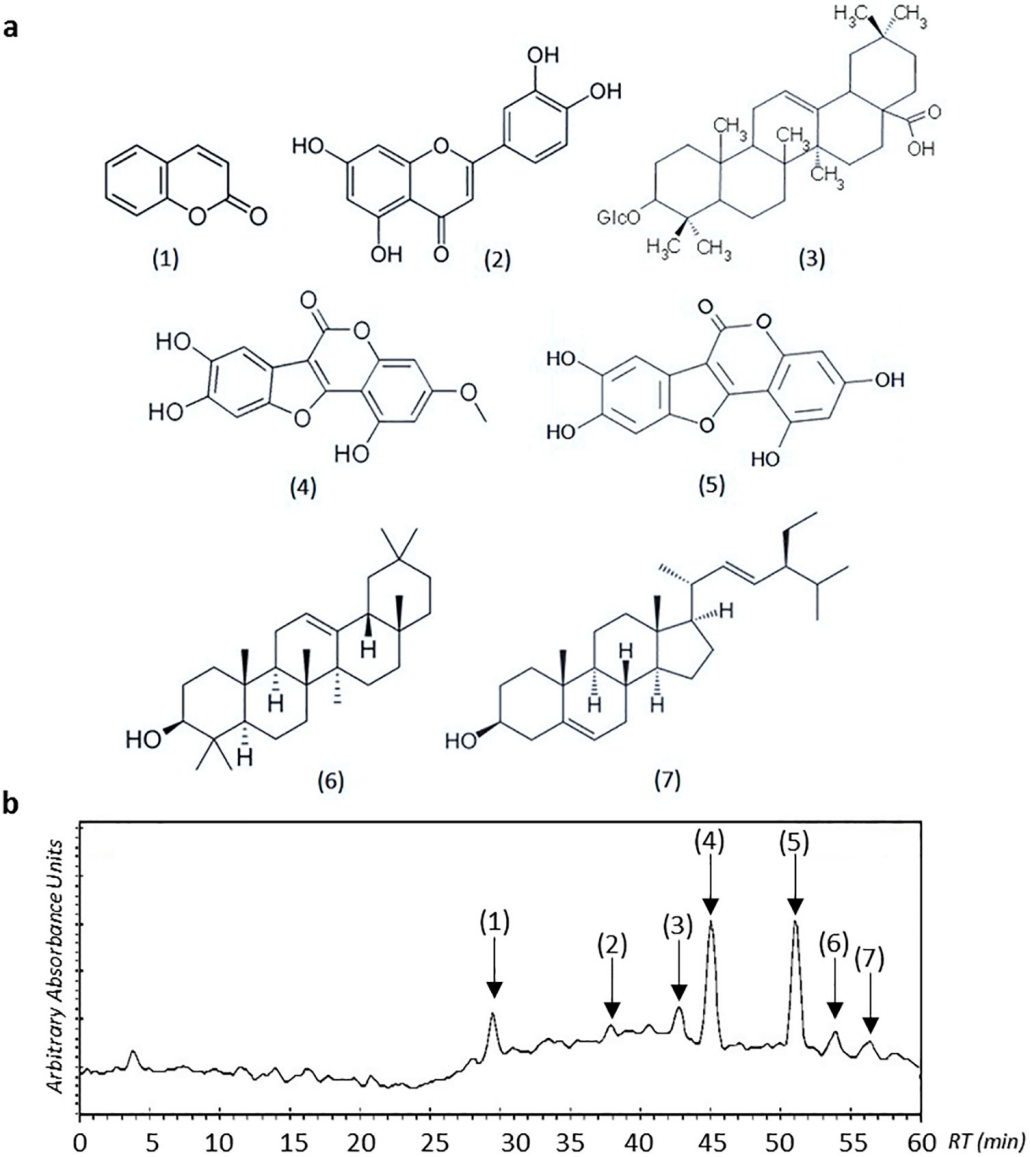
a) Structures of the main phytochemicals detected at 204 nm in methanolic extract of *E*. *alba* callus [(1): Coumarin; (2): Luteolin; (3): Eclalbatin; (4): demethylWedelolactone; (5): Wedelolactone; (6): β-amyrin; (7): Stigmasterol]; b) Typical HPLC chromatogram recorded at 204 nm of methanolic extract of *E*. *alba* callus.

*E*. *alba* contains some of the major phytochemicals including triterpenes, coumestans, polyacetylenes, polypeptides and flavonoids [11, 67]. Quantification of these precious compounds in callus culture of *E*. *alba*, placed under the influence of monochromatic lights, was carried out using high performance liquid chromatography (HPLC). A typical HPLC chromatogram depicting the main phytochemicals detected at 204 nm in methanolic extract of *E*. *alba* callus is presented in Fig 6. All of these compounds were recorded highest in callus culture exposed to red light except stigmasterol, which was found higher under blue light (0.22 mg/g) (Table 2). Younas et al. [45] reported highest silymarin accumulation in *S*. *marianum* callus culture in response to red light. Similar results were documented by Kuo et al. [68] in cucurbitacin accumulation under red light using *in vitro* cultures. On the contrary, Khan et al. [60] and Nadeem et al. [49] showed maximum phytochemical production under white and blue light in *Fagonia indica* and *Ocimum basilicum* callus cultures, respectively. Major constituents of secondary metabolites were appeared to be eclalbatin, wedelolactone and dimethyl wedelolactone as previously reported by Diogo et al. [69] (Table 2). Phytochemical quantification showed negative effect of yellow light on levels of major phytochemicals compared to control treatment which is in contrast to study of Victório et al. [70], stating the stimulating effects of yellow light for phenolics production. These findings suggested that influence of light on plant cells varies largely, depending upon plant species, light quality, intensity and culture type.

**Table 2:** Secondary metabolites accumulation under multi spectral lights in callus culture of *E*. *alba*. Values represent means ± standard errors from triplicates.

Lights Treatment	Phytochemical Profile of *Eclipta alba* (mg/g DW)						
	B-Amyrin	Stigmasterol	Luteolin	Coumarin	Eclalbatin	Wedelolactone	dmWedelolactone
**Control**	0.09 ± 0.004	0.07 ± 0.003	0.18 ± 0.002	0.41 ± 0.03	3.15 ± 0.84	20.50 ± 1.05	14.92 ± 1.95
**Green**	0.08 ± 0.006	0.07 ± 0.001	0.25 ± 0.006	0.65 ± 0.01	3.64 ± 0.22	23.67 ± 0.94	14.52 ± 1.37
**Yellow**	0.07 ± 0.001	0.12 ± 0.02	0.22 ± 0.003	0.40 ± 0.09	2.43 ± 0.39	15.81 ± 1.73	10.74 ± 0.11
**Red**	0.38 ± 0.003	0.11 ± 0.009	0.39 ± 0.008	1.26 ± 0.04	5.00 ± 0.83	32.54 ± 1.04	23.67 ± 1.29
**Blue**	0.15 ± 0.008	0.22 ± 0.03	0.30 ± 0.003	0.92 ± 0.03	4.52 ± 0.94	29.38 ± 1.68	19.24 ± 1.38
**Dark**	0.09 ± 0.002	0.17 ± 0.008	0.34 ± 0.001	1.10 ± 0.07	4.92 ± 0.37	32.05 ± 2.46	21.09 ± 1.06
**White Light** (24h)	0.23 ± 0.005	0.16 ± 0.007	0.30 ± 0.005	0.85 ± 0.04	4.05 ± 0.41	26.35 ± 1.62	17.34 ± 0.94

The present work, using Pearson correlation coefficient calculation, allowed to correlate certain biological activities with some specific metabolites of the methanolic extract of *E*. *alba* callus and the biological activities (Table 3). Eclalbatin and demethylwedelolactone were linked to the DPPH radical scavenging activity, stigmasterol to the ABTS antioxidant capacity, whereas all these phytochemicals, with the exception of β-amyrin were correlated with FRAP antioxidant capacity (Table 3). Eclalbatin and demethylwedelolactone accumulation correlated with the *in vitro* inhibition of the formation of both type of advanced glycation end products (Table 3). Stigmasterol was significantly associated with α-glucosidase inhibition capacity of the extract, while both eclalbatin and demethylwedelolactone contents correlated with α-amylase inhibition (Table 3). Lipase inhibition was correlated with luteolin, eclalbatin and demethylwedelolactone accumulations (Table 3). Future works using *in cellulo* and/or *in vivo* animal models will be considered in the future to confirm these trends.

**Table 3:** Correlation analysis between phytochemicals and biological activities using Pearson correlation coefficient (PCC).

Variables	1	2	3	4	5	6	7
DPPH	0.708	0.632	0.801*	0.800*	0.701	0.499	0.401
ABTS	0.537	0.496	0.608	0.606	0.488	0.352	0.754*
FRAP	0.769*	0.778**	0.747*	0.746*	0.652	0.547	0.764*
α-glucosidase	0.624	0.645	0.609	0.608	0.452	0.266	0.815*
α-amylase	0.679	0.683	0.672*	0.671*	0.569	0.507	0.725
Lipase	0.725	0.756*	0.677*	0.676*	0.586	0.562	0.750
vesp-AGE	0.709	0.722	0.687*	0.686*	0.586	0.519	0.734
pent-AGE	0.708	0.721	0.686*	0.685*	0.586	0.519	0.734

(1): Coumarin; (2): Luteolin; (3): Eclalbatin; (4): demethylWedelolactone; (5): Wedelolactone; (6): β-amyrin; (7): Stigmasterol; Significance level:

* p < 0.05;

** p < 0.01;

*** p < 0.001.

## Conclusions

In conclusion, differential influence of light quality was evaluated on *in vitro* derived callus cultures of *Eclipta alba* L. under multispectral lights. After 28 days exposure, calli were harvested and analyzed for biomass production, phytochemical synthesis and biological activities. Maximum dry weight accumulation was observed under red light compared to rest of treatments. Furthermore, red light significantly triggered phenolics and flavonoids biosynthesis in callus culture of *E*. *alba*. HPLC quantification showed highest accumulation of major secondary metabolites such as eclalbatin, wedelolactone and dimethyl wedelolactone under red light whereas blue light significantly enhanced stigmasterol level in *E*. *alba*. Antioxidant, lipase and antidiabetic activities of calli extracts were found elevated in red light compared to control. A positive correlation in metabolites biosynthesis and their respective biological activities was observed.
